# Two Isoforms of the mRNA Binding Protein IGF2BP2 Are Generated by Alternative Translational Initiation

**DOI:** 10.1371/journal.pone.0033140

**Published:** 2012-03-12

**Authors:** Hang T. T. Le, Alice M. Sorrell, Kenneth Siddle

**Affiliations:** University of Cambridge Metabolic Research Laboratories and Department of Clinical Biochemistry, Institute of Metabolic Science, Addenbrooke's Hospital, Cambridge, United Kingdom; Vanderbilt University Medical Center, United States of America

## Abstract

IGF2BP2 is a member of a family of mRNA binding proteins that, collectively, have been shown to bind to several different mRNAs in mammalian cells, including one of the mRNAs encoding insulin-like growth factor-2. Polymorphisms in the *Igf2bp2* gene are associated with risk of developing type 2 diabetes, but detailed functional characterisation of IGF2BP2 protein is lacking. By immunoblotting with C-terminally reactive antibodies we identified a novel IGF2BP2 isoform with a molecular weight of 58 kDa in both human and rodents, that is expressed at somewhat lower levels than the full-length 65 kDa protein. We demonstrated by mutagenesis that this isoform is generated by alternative translation initiation at the internal Met69. It lacks a conserved N-terminal RNA Recognition Motif (RRM) and would be predicted to differ functionally from the canonical full length isoform. We further investigated IGF2BP2 mRNA transcripts by amplification of cDNA using 5′-RACE. We identified multiple transcription start sites of the human, mouse and rat *Igf2bp2* genes in a highly conserved region only 50–90 nts upstream of the major translation start site, ruling out the existence of N-terminally extended isoforms. We conclude that structural heterogeneity of IGF2BP2 protein should be taken into account when considering cellular function.

## Introduction

Mammalian IGF2 mRNA binding proteins (IGF2BPs or IMPs), also known as VICKZ proteins (for **V**g1-RBP/Vera, **I**MP, **C**RD-BP, **K**OC, **Z**BP-1), are proteins of ∼65 kDa containing two N-terminal RNA recognition motifs (RRMs) and four hnRNP K-homology (KH) domains [1]. RRMs [2] and KH domains [3] function as RNA binding modules in diverse proteins, and also participate in protein-protein interactions including dimer formation. All three IGF2BPs bind to at least 6 sites on IGF2 leader 3 mRNA [1] and to a site within the 3′-UTR of IGF2 mRNAs [4]. However, each of the IGF2BPs has been independently identified in other contexts (reviewed in [5], [6]): IGF2BP1 is orthologous to chicken ZBP-1 and mouse CRD-BP, which have been implicated in sorting β-actin mRNA and stabilizing c-myc mRNA respectively; IGF2BP2 is a splice variant of a p62 protein identified as an autoantigen in hepatocellular carcinoma; IGF2BP3 is identical to the KOC protein over-expressed in pancreatic cancer, and orthologous to Xenopus Vg1-RBP implicated in mRNA trafficking. Studies on IGF2BPs -1 and -3 have indicated diverse mRNA targets, which lack a common well-defined recognition motif [6], [7]. Indeed, over 300 different mRNAs were identified in IGF2BP1-containing ribonucleoprotein-containing granules in HEK293 cells, among which transcripts encoding proteins involved in protein secretion and metabolism were highly represented [8]. IGF2BPs have been reported to influence the stability and localization of target mRNAs, and to act as both inhibitors and activators of their translation, depending on the sequences examined and cellular context [1], [7], [9], [10], [11], [12], [13]. Importantly, differences in activity of individual IGF2BPs towards specific mRNAs have been clearly demonstrated [11].

The physiological roles of IGF2BPs are as yet unclear [1], [6], [14]. In mice, all three IGF2BPs are highly expressed in the embryo, peaking around E12.5 and declining towards birth, with low or undetectable levels in most adult tissues [1] although high levels have been observed in many solid tumours. Transgenic over-expression of IGF2BP1 in mice induced a high level of mammary tumours [15], while targeted inactivation of the *Igf2bp1* gene resulted in growth retardation and impaired gut development [9]. Transgenic over-expression of IGF2BP3 caused subtle morphological alterations in the pancreas [16], and loss of function analysis in Xenopus embryos indicated that the IGF2BP3 orthologue Vg1-RBP is required for establishment of pancreatic fate within the endoderm [17]. Polymorphisms within intron 2 of the *Igf2bp2* gene influence type 2 diabetes risk [18], [19], [20], [21] and have been linked to reduced early phase insulin release and other indices of impaired pancreatic beta cell function [22], [23], [24]. Another polymorphism in the promoter region of the *Igf2bp2* gene has been linked to adiposity, and hence insulin resistance [25]. Although it has not been conclusively established that polymorphisms within the *Igf2bp2* gene affect diabetes susceptibility through changes in the activity of IGF2BP2 protein per se [26], it is highly plausible that IGF2BP2 might influence the development and/or function of the pancreas or adipose tissue through effects on the expression of IGF2 or other proteins [14].

It would be expected that the specificity and functional consequences of mRNA binding might differ between isoforms of a given IGF2BP as well as between family members. A p62 splice variant of human IGF2BP2 has been identified, which lacks exon 10 encoding 43 amino acids between the KH2 and KH3 domains [27]. There is no experimental evidence for analogous splice variants of rodent IGF2BP2s, nor of human IGF2BPs -1 and -3, but public databases predict multiple mRNA transcripts encoding distinct isoforms of all three human IGF2BPs (ENSG00000159217, ENSG00000073792, ENSG00000136231) and of rodent IGF2BP2 (ENSMUSG00000033581, ENSRNOG00000025946). Moreover, in rat the genomic sequence contains an open reading frame of 627 nts upstream of the methionine codons corresponding to the translation initiation sites for human and mouse IGF2BP2, compatible with the existence of an N-terminally extended isoform. Transcription start sites have not been experimentally determined, but it has been reported that mRNA encoding the p62 variant of human IGF2BP2 has a 5′-UTR of 435 nts [27].

We observed by Western blotting that IGFBPs -1 and -3 are each expressed only as a single isoform in human, mouse and rat tissues and cell lines, but for IGF2BP2 two isoforms were present. We show here that these IGF2BP2 isoforms arise as a consequence of translation initiation from different sites, rather than alternative splicing, such that the smaller isoform lacks an N-terminal RNA recognition motif. We also show that in human, mouse and rat the 5′UTR of ∼50–90 nts relative to the canonical translation initiation site lacks an upstream open reading frame, ruling out the occurrence of N-terminally extended isoforms.

## Results

### Identification of IGF2BP2 transcription initiation sites using 5′RLM-RACE

IGF2BP2 sequences on online databases such as NCBI and Ensembl show significant discrepancies at the 5′ ends. However, no experimental data on the transcription start site of IGF2BP2 have been published, except that of the human p62 splice variant for which the transcription start site was reported to be 435 nt upstream of the translation initiation codon [27]. To determine experimentally the site(s) of transcription intiation, 5′ RNA ligase-mediated rapid amplification of cDNA ends (5′ RLM-RACE) was performed. This method offers a major advantage over traditional 5′RACE in that it amplifies cDNA only from full-length, capped mRNA, therefore allowing identification of the actual 5′ ends of mRNAs. Total RNA was first treated with Calf Intestine Alkaline Phosphatase (CIP) to remove 5′ phosphate from non-mRNAs and incomplete mRNAs. Samples were then treated with Tobacco Acid Pyrophosphatase (TAP) to remove the cap structure of intact mRNAs, leaving a 5′ phosphate group on this mRNA subset only, followed by ligation of an RNA adapter to the decapped mRNAs. Reverse transcription and subsequent PCR amplification using gene-specific and adapter-specific primers allows the 5′ ends of mRNA transcripts to be mapped.

**Figure 1:**
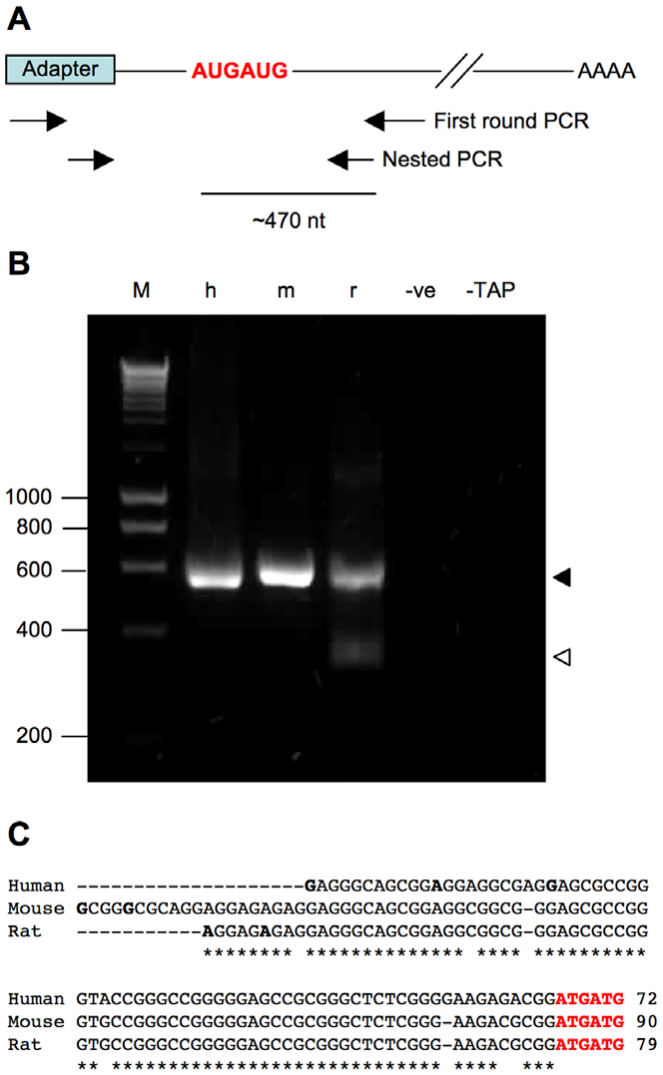
Identification of transcription initiation site of IGF2BP2 by 5′RLM-RACE. Total RNA from human HEK293 fibroblasts, mouse 3T3-L1 preadipocytes and rat placenta was used as templates for the RACE experiment. (A) Schematic representation of 5′RLM-RACE PCR. Two forward primers recognizing the adapter sequence, and two reverse primers against exon 6 of human/mouse/rat IGF2BP2 are shown. The inner primer is 467 nt downstream of the canonical translational start site in mouse and rat, and 473 nt downstream in human. (B) Agarose gel electrophoresis of nested PCR reaction products. Molecular size markers (base pairs) are indicated on the left. The correct product is indicated by a filled triangle, and the non-specific product is indicated by an open triangle. M: marker, h: human, m: mouse, r: rat, −ve: PCR negative control using water as template, −TAP: negative control using RNAs that was not treated with TAP, therefore could not ligate to the RNA adapter. (C) Sequence alignment of human, mouse and rat 5′UTR as identified by 5′RLM-RACE. The transcription start sites are in bold and underlined. The canonical translation initiation site is shown in red. The position of the most 5′ transcription start site relative to the translation initiation site is indicated at the end of the sequences.

5′RLM-RACE was performed using total RNA from human HEK293 fibroblasts, mouse 3T3-L1 preadipocytes and rat placenta, all of which had previously been demonstrated by RT-PCR and Western blotting to express IGF2BP2 at moderately high levels (data not shown). The first round of 5′RACE-PCR was carried out with a forward outer primer corresponding to the adapter and a reverse primer specific for IGF2BP2. Second round nested PCR used the first round PCR products as templates, and two inner primers recognising the adapter and IGF2BP2 sequence. The two IGF2BP2-specific primers were designed in two regions in exon 6 with 100% sequence identity between human, mouse and rat so that they could be used for samples from all three species. In human and mouse, a single band was obtained after the 5′RACE nested PCR reaction (Figure 1B, lane 2 and 3) with estimated size of ∼560 bp. Meanwhile, two bands were observed in rat (lane 4), one of similar size to the human and mouse samples and the other of lower molecular weight of ∼370 bp. Gel purification, cloning and sequencing of this lower band revealed that it was a non-specific product. Cloning and sequencing of the ∼560 bp bands from all three samples mapped the transcription start site of IGF2BP2 to a highly conserved region 51–90 nt upstream of the translation initiation site (Figure 1C). At least two transcription start sites were identified for each species. In human, three sites were positioned 51, 65 and 72 nt upstream of the double Met start codon. In mouse and rat, the two sites were 86 and 90 nt, and 74 and 79 nt upstream of the presumed translational initiator codon, respectively.

### IGF2BP2 is expressed in two distinct isoforms

**Figure 2:**
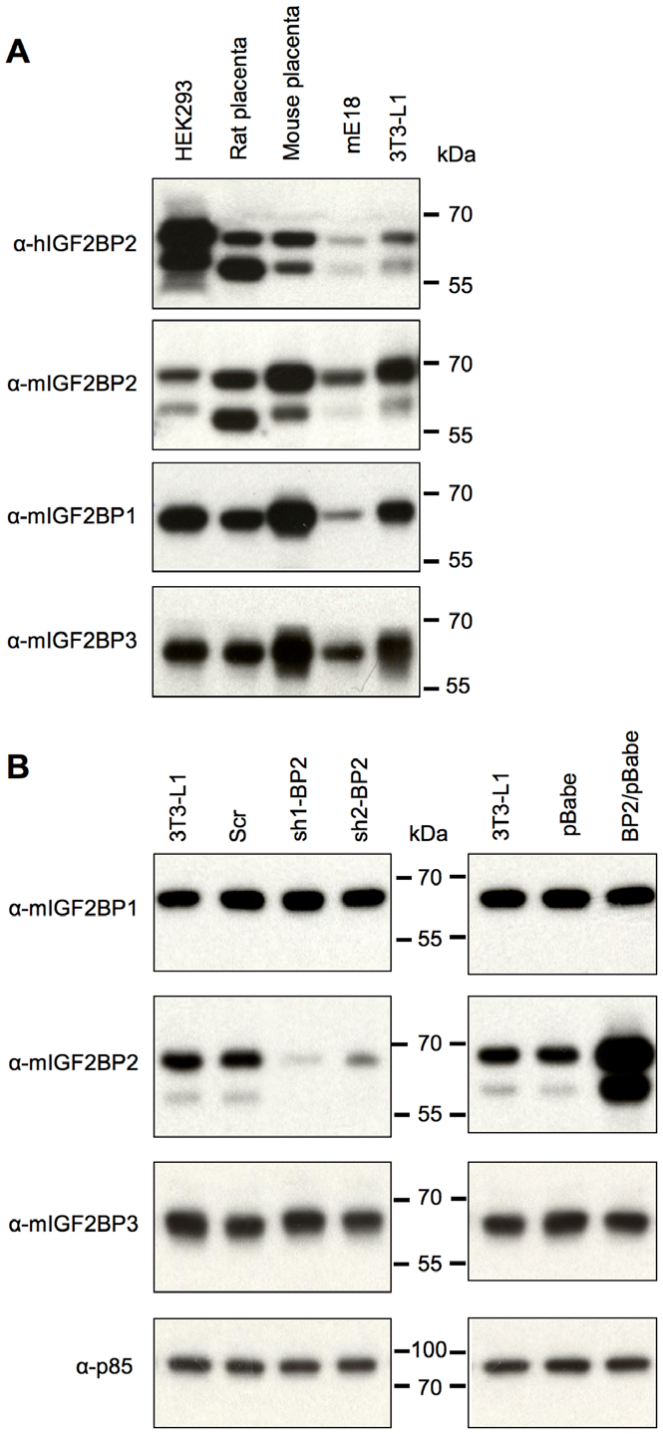
Detection of a novel IGF2BP2 isoform *in vivo*. **A.** Expression of endogenous IGF2BP1/2/3 expression in human, rat and mouse. Samples (10 µg of total protein) from human HEK293 cells, rat placenta, mouse placenta, mouse embryo E18 (mE18) and 3T3-L1 cells were resolved by 10% SDS-PAGE and analysed by Western blot using an antibody against an internal fragment of human IGF2BP2 (Abnova) (panel 1) and antibodies against the C-terminus of mouse IGF2BP2/1/3 (CRB Ltd) (panels 2, 3 and 4). **B.** Knockdown and overexpression of IGF2BP2 in 3T3-L1 preadipocytes using retrovirus. 3T3-L1 cells were treated with control scrambled shRNA (Scr) or two independent shRNA constructs targeting endogenous IGF2BP2 (sh1-BP2 and sh2-BP2) (left panels). An empty control vector (pBabe) and a vector containing IGF2BP2 cDNA sequence (BP2/pBabe) were also introduced into 3T3-L1 cells to overexpress the protein (right panels). Samples (10 µg of total protein) from human HEK293 cells, rat placenta, mouse placenta, mouse embryo E18 (mE18) and 3T3-L1 cells were resolved by 10% SDS-PAGE and analysed by Western blot using antibodies against the C-terminus of mouse IGF2BP2/1/3 (CRB Ltd) (panels 1, 2 and 3). In both experiments untreated 3T3-L1 cells are in the left-most lane. Positions of molecular weight markers are indicated.

The full length human and mouse IGF2BP2 proteins are comprised of 599 and 592 aa respectively, with a molecular mass of ∼66 kDa [1], [28]. Analysis of endogenous IGF2BP2 by immunoblotting unexpectedly revealed two distinct isoforms of IGF2BP2 in human, mouse and rat, one at the predicted molecular weight of ∼66 kDa and a smaller species at ∼58 kDa (Figure 2A, panels 1 and 2). This result was observed with two different antibodies, one directed against the C-terminus of mouse IGF2BP2 and one against an internal epitope of human IGF2BP2. Only a single band was observed for both IGF2BP1 and IGF2BP3 (Figure 2A, panels 3 and 4). Two different, specific shRNA constructs against IGF2BP2 suppressed the expression of both species in 3T3-L1 cells, while over-expression of transfected IGF2BP2 cDNA generated both species (Figure 2B), indicating that they were derived from IGF2BP2 mRNA(s).

**Figure 3:**
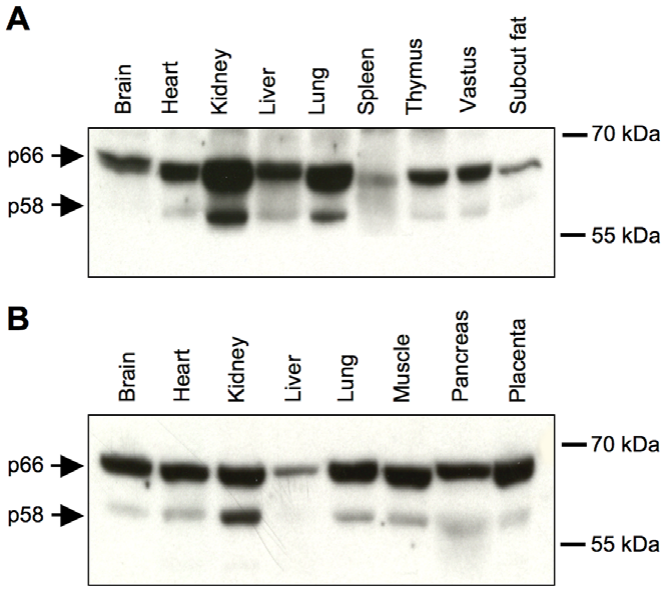
Tissue distribution of IGF2BP2 isoforms. Expression of IGF2BP2 was analysed by Western blotting in tissues from 3-day old rat (**A**) and E19 mouse embryo (**B**). Total cellular proteins in lysates prepared from the tissues indicated (25 µg/lane) were resolved by 10% SDS-PAGE and blots were probed with antibody to IGF2BP2 C-terminal peptide (CRB Ltd). Positions of molecular weight markers are indicated to the right.

It has previously been reported that expression of all three IGF2BPs is high in embryos but very low in most adult human and rodent tissues [1]. We therefore examined the expression of IGF2BP2 isoforms in perinatal rodent tissues (Figure 3). The 58 kDa protein was detected as a minor isoform except in tissues where the overall expression was comparatively low. Expression of p58 relative to p66 was higher in kidney and lower in brain compared to other tissues examined.

In human, it is well known that alternative splicing of IGF2BP2 results in a transcript lacking the short exon 10 and encoding a p62 protein [29]. Recently, an AT-rich regulatory region responding to the architectural transcription factor HMGA2 has been identified in the first intron of the IGF2BP2 gene, and this has been suggested potentially to function as part of an alternative promoter directing the transcription of a shorter IGF2BP2 transcript lacking exon 1 [30]. However, it is unlikely that the 58 kDa isoform was produced by either alternative splicing or transcription under an alternative promoter in intron 1 since this isoform could be generated from the cloned cDNA coding sequence of IGF2BP2. This indicates that the small isoform is generated post-transcriptionally, either by protease cleavage from full-length p66 isoform or by alternative translation initiation via an Internal Ribosomal Entry Site (IRES) or leaky ribosomal scanning. Post-translational modifications such as glycosylation and phosphorylation should make the protein migrate more slowly on SDS-gels, and are therefore unlikely to account for the smaller band on Western blot.

### Identification of Met69 as the putative alternative translation initiation site

**Figure 4:**
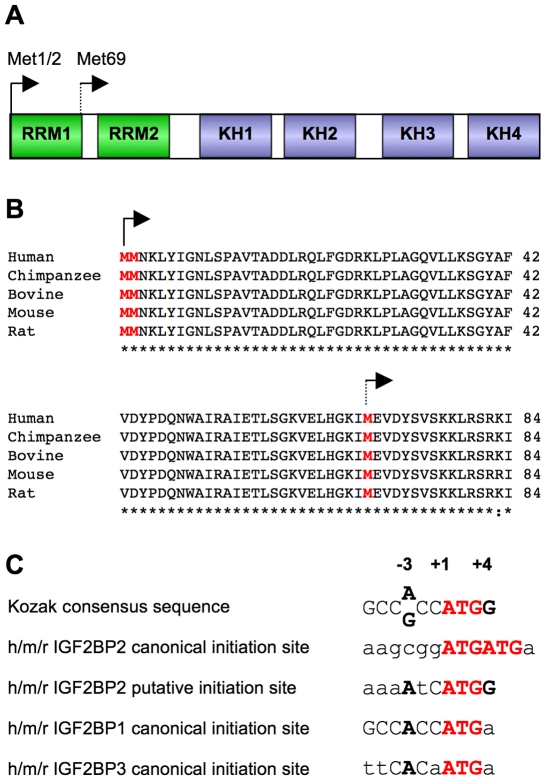
Identification of Met69 as the putative alternative translation initiation site. **A.** Schematic representation of IGF2BP2 and locations of the canonical initiation site at Met1/2 (solid arrow) and the putative alternative translational initiation site at Met69 (broken arrow). **B.** Protein sequence alignment of the 5′ region of IGF2BP2 in human, chimpanzee, cow, mouse and rat,. Two putative translational initiation sites are shown by arrows with solid and broken lines, respectively. **C.** Nucleic acid sequence alignment of IGF2BP1/2/3 translational initiation sites and the surrounding sequences. Bases that match the consensus are in upper case, while those that do not match the consensus are in lower case. The start codons are in red, and the most two important positions in the Kozak consensus are in bold.

An inspection of IGF2BP2 mRNA sequence reveals a plausible alternative initiation codon corresponding to the first internal Met downstream of the canonical double Met initiation site. Translation initiation at this internal Met69 would generate a N-terminally truncated product lacking the first RRM domain (Figure 4A), with a predicted molecular weight of ∼58 kDa, which matches the molecular weight of the smaller isoform seen on Western blot. Both nucleotide sequence and protein sequence around this internal Met69 are highly conserved in various species (Figure 3B).

Examination of the IGF2BP2 gene sequence also reveals a poor Kozak consensus [31] around the canonical AUG start codon while the downstream Met69 is in a strong Kozak context (Figure 4C), suggesting leaky scanning as the most plausible mechanism to generate the small p58 isoform.

### The small IGF2BP2 isoform is generated by leaky ribosomal scanning

**Figure 5:**
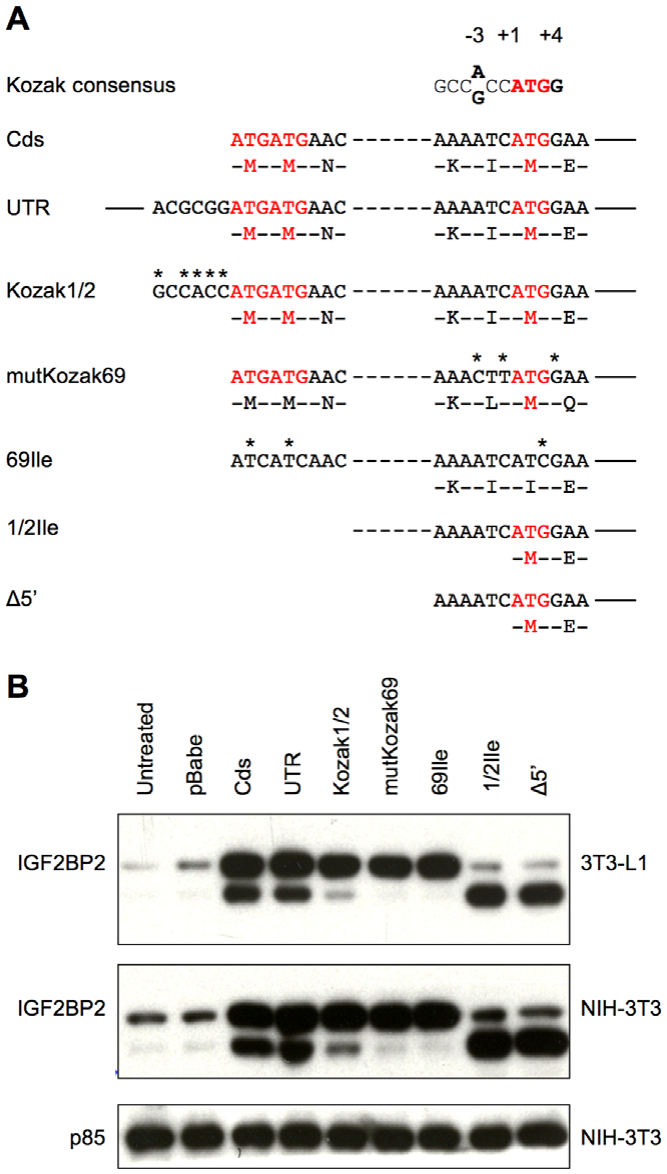
The p58 isoform of IGF2BP2 is generated by leaky ribosomal scanning. **A.** A schematic representation of mIGF2BP2 mutants. Only the sequences surrounding the canonical translation initiation site and the internal Met69 are shown. The two putative translation initiation sites are in red. *Cds*, wild-type coding sequence of IGF2BP2; *UTR*, wild-type IGF2BP2 with 34 nt 5′UTR and 336 nt 3′UTR sequences included; *Kozak1/2*, wild-type IGF2BP2 with added Kozak sequence upstream of the canonical starting codon, *1/2Ile*, the first and second ATG codon was mutated to ATC; *69Ile*, the internal Met69 codon was mutated to ATC, *mutKozak69*, the Kozak consensus sequence flanking the internal Met69 was mutated; *Δ5′*, the first 66 codons including the first and second ATG codons were deleted. **B.** Protein analysis of mouse IGF2BP2 mutants expressed in 3T3-L1 preadipocytes (upper panels) and NIH-3T3 fibroblasts (lower panels) using retrovirus vectors. Untreated cells, and cells transfected with the empty vector pBabe, were used as controls. Cellular proteins (10 µg/lane) were resolved by 10% SDS-PAGE and blots were probed with antibody to IGF2BP2 C-terminal peptide (CRB Ltd) and with antibody to the p85 subunit of PI3-kinase as a loading control.

To test whether the 58 kDa isoform of IGF2BP2 is the product of alternative translational initiation from the internal Met69, various mutagenised constructs were made (Figure 5A), in which the presumed initiator double Met1/2, the internal Met69 and/or their flaking sequences were mutated, so as to enhance or diminish translation initiation at one or other of these sites.

Expression from the coding sequence alone resulted in production of the two isoforms, as did the constructs with fragments of 5′ and 3′ UTR sequences (Figure 5B, lanes 3, 4). When a stronger Kozak sequence was added before the canonical start codon, the level of the small isoform was significantly reduced (Figure 5B, lane 5). It should be noted that this is still only a medium-strength Kozak sequence as the A nucleotide at position +4 was not changed to a G as in the optimal consensus. Mutation of the Kozak consensus surrounding the internal Met69 eliminated production of the small isoform (Figure 5B, lane 6). Similarly, when the internal Met69 was mutated to Ile, only the large isoform was detected (Figure 5B, lane 7), whereas mutation of the double Met1/2 caused only the small isoform to be produced (Figure 5B, lane 8). Additionally the N-terminally truncated IGF2BP2 construct which was designed to start at the internal Met69 gave rise to only the small isoform (Figure 5B, lane 9). These results strongly support the hypothesis that the small IGF2BP2 isoform is produced by alternative translation initiation via leaky ribosomal scanning at the internal Met69.

**Figure 6:**
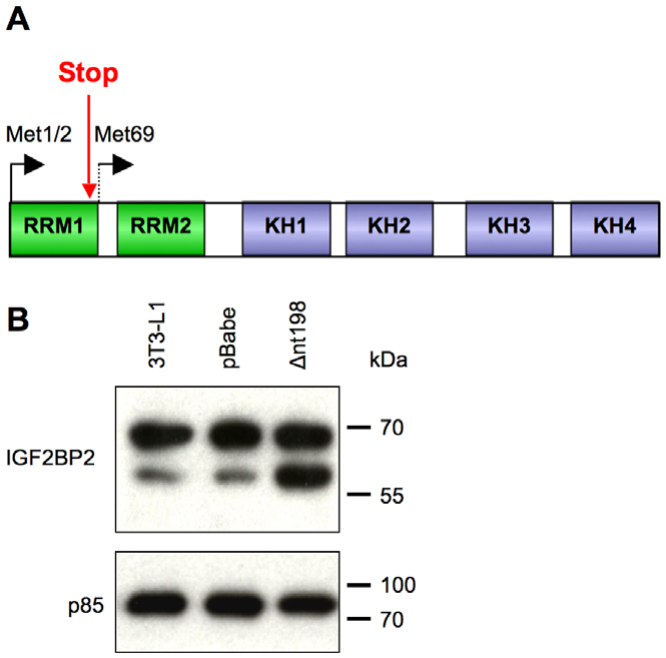
The p58 isoform of IGF2BP2 is not generated by protease cleavage. **A.** A schematic representation of the frameshift mutation. A single nucleotide deletion at position 198 resulted in a premature stop codon in the ORF starting at the double Met but did not affect the downstream ORF starting from the internal Met69. A Kozak sequence was also added upstream of the canonical double Met. **B.** Western blot analysis of the frameshift construct expressed in 3T3-L1 preadipocytes. Empty vector (pBabe) or the frameshift construct (Δnt198) was introduced into the cells using retrovirus. Cellular proteins (10 µg/lane) were resolved by 10% SDS-PAGE and blots were probed with antibody to IGF2BP2 C-terminal peptide (CRB Ltd) and with antibody to the p85 subunit of PI3-kinase as a loading control. Positions of molecular weight markers are indicated to the right.

If the smaller isoform was generated by IRES-mediated initiation, then changing the sequence flanking the canonical double Met1/2 initiation site should not affect production of the p58 isoform. In fact, the expression level of the p58 isoform was significantly decreased after addition of a Kozak consensus upstream of Met1/2 (Figure 5B, lane 5), allowing us to exclude IRES-mediated initiation as the source of IGF2BP2 small isoform. The p58 isoform is also unlikely to be a product of N-terminal proteolytic cleavage of the full-length p66 isoform, based on its level of expression from this construct. The Kozak1/2 construct contained no modification in the coding sequence, and had the smaller isoform been generated from proteolysis its relative expression compared to the long isoform should have not been affected, which was not the case as in seen Figure 5B, lane 5. To completely rule out proteolysis as an explanation for generation of the smaller isoform, a single deletion was introduced in IGF2BP2 cDNA at nt 198, upstream of Met69, causing a frameshift and premature termination (Figure 6A). A Kozak sequence was also added upstream of Met1/2 to drive expression from this canonical site. This disturbance affected neither the ORF starting from the internal Met69 nor the sequence context surrounding this internal Met. From this frameshift construct (Δnt198), the small isoform was still generated despite the fact that no full-length isoform was expressed (Figure 6B). Its level of overexpression relative to the endogenous protein matched that of the Kozak1/2 construct (lane 5, Figure 5B), due to more efficient translation initiation at the canonical double Met. This experiment allows us to exclude proteolytic cleavage as the main source of the p58 isoform, and confirm that the small isoform is generated by alternative translation initiation from the in-frame Met69 via leaky ribosomal scanning.

## Discussion

The *Igf2bp2* gene has been identified as a type 2 diabetes susceptibility locus [18], [19], [20], [21]. However, rather little is known about the structure and function of the IGF2BP2 protein, and the manner in which its activity might influence diabetes risk. In this study, we identified the transcription start sites in human, mouse and rat *Igf2bp2* genes, and showed that two isoforms of the IGF2BP2 protein are generated as a result of alternative translation initiation at the internal Met69.

To determine the transcription start site (TSS) of *Igf2bp2*, we carried out 5′RLM-RACE. Multiple TSSs were identified in both human and rodents, which all mapped to a highly conserved region 51–90 nt upstream of the canonical translation initiation site of IGF2BP2. This is considerably shorter than the 435 nt 5′UTR of the human splice variant p62 previously reported [27]. However a BLAST of this 435 nt sequence against the human genome reference sequence revealed that only the first 62 nts upstream of the canonical translation initiation site match the human *Igf2bp2* sequence. The remaining 373 nts of this reported 5′UTR are identical to TSC22 domain family member 1, isoform 1 which is located on human chromosome 13, while human IGF2BP2 is located on chromosome 3 at 3q27.2. Therefore the long 5′UTR reported for p62 was a cloning artefact. The 5′UTR of human IGF2BP2 included in the reference sequences (NM_001007225 and NM_006548) is 79 nts. A previous study involving 5′ RLM-RACE analysis of RNA isolated from murine NIH-3T3 fibroblasts [30] found the most 5′ TSS of IGF2BP2 was 84 nt upstream of the translation start site, and 29 nt downstream of the most 5′ TSS obtained from the Database of Transcription Start Sites (http://dbtss.hgc.jp/), similar to our results. It is well-known that in mammals, transcription does not initiate at a single site, but rather at multiple sites across a small region, and that alternative TSS usage depends on tissue, CpG islands, promoter structures and imprinting [32]. In this study, we only sequenced a small number of clones from 5′RACE-PCR products, six for each species, and identified 2–3 TSSs. It remains possible that other less common *Igf2bp2* transcriptional start sites might be utilised under certain conditions or in other cell types.

The rat genomic *Igf2bp2* sequence contains an open reading frame of 627 nts upstream of the double Met codons which correspond to translation initiation sites for human and mouse IGF2BP2. Identification of TSSs less than 100 nt upstream of the double Met codons in rat rules out the existence of longer transcripts formerly annotated as rat IGF2BP2 mRNA sequences. The mouse genomic sequence is homologous to that of the rat over this region, and also contains the upstream AUG codon, but the presence of two in-frame stop codons downstream eliminates potential expression from this uORF. The human and rodent genomic sequences diverge ∼270 nt upstream of the canonical translation initiation site, and no equivalent sequence to the rodent uORF is found in human. Since the TSS of rat as identified in this study is in a region highly homologous to that of human and mouse, we believe the translation initiation site of rat *Igf2bp2* is at the double Met, as in the other species. Moreover, Western blot of rat placenta did not detect any immunoreactive proteins of ∼800 amino acids, such as would be generated from the upstream AUG. However, given the existence of an extended in-frame uORF in the rat genomic sequence, the possibility cannot be completely ruled out that larger isoforms of rat IGF2BP2 might be generated in a tissue-specific manner by alternative promoter usage.

Although Western blotting with a C-terminally reactive antibody provided no evidence for isoforms of IGF2BP2 larger than the canonical 66 kDa protein in rodents or humans, it did reveal the presence of a novel smaller isoform of ∼58 kDa in all species. As demonstrated by site-directed mutagenesis, the small isoform is produced by alternative translational initiation at Met69. This isoform thus lacks the first 68 amino acids corresponding to the RRM1 domain. The relative expression of 66 kDa and 58 kDa isoforms showed modest species and tissue differences. The long isoform was consistently the more abundant although the ratio of short/long isoform was higher in kidney and lower in brain compared to other tissues examined. These observations leave open the possibility that relative expression of the two isoforms might be subject to regulation.

Alternative translation initiation has long been recognised and is not uncommon [33]. In some instances it is used as a mechanism to generate protein isoforms with different intracellular localization, expression patterns or physiological functions [34], [35], [36]. There is no obvious localization signal in the RRM1 domain of IGF2BP2, and deletion of the two RRM domains in the homolog IGF2BP1 has been shown to have no effect on sub-cytoplasmic localization of the protein in NIH3T3 cells [37]. The relative protein expression of the two IGF2BP2 isoforms in different tissues, at different stages of development or under different physiological conditions is difficult to study as the protein is expressed at very low levels in most adult tissues except gonads [38]. It is interesting that isoforms of the RDM1 protein showed differing responses to heat shock dependent on RRM domains. While the expression of long N-terminal isoforms with intact 5′ RRM domain remained unchanged, N-terminal truncated isoforms lacking this domain had their expression modulated after heat shock [39]. It is therefore possible that the two isoforms of IGF2BP2 could be differentially regulated by external stimuli.

The first RRM domain of IGF2BP2, which is absent in the small isoform, contains highly conserved RNP1 and RNP2 motifs important for RNA recognition, in contrast to the second RRM domain which has poor RNP signatures [2], [40]. In addition to binding to RNA, RRM domains can participate in protein-protein interaction and facilitate inter- or intra-molecular dimerisation of RRM domain-containing proteins [2], [40]. Thus loss of the RRM1 domain in the small isoform might affect its binding affinity and specificity to both RNA and protein partners, as well as its own structure, dimer formation, and stability as seen in other RRM-containing proteins such as hnRNP Q, PARN and nPTB [41], [42], [43]. In the case of the closely related IGF2BP1, a construct lacking both RRM domains bound H19 RNA with 5-fold lower affinity than the full-length protein, although the RRM1/2 di-domain did not itself bind this RNA in a mobility-shift assay [37]. Thus RRM domains in IGF2BPs may function to modulate the RNA-binding activity of the KH domains.

In conclusion, we demonstrate here the complexity of *Igf2bp2* gene expression. Transcription starts at multiple sites in a short region highly homologous in human and rodents to generate transcripts with a relatively short 5′UTR (60–90 nts). Alternative translation initiation at the canonical double Met1/2 and the internal Met69 generates two isoforms of IGF2BP2 protein. Further study on functional differences between the two isoforms may provide insight into how cells regulate the expression and activity of these two isoforms and may shed light on how polymorphisms in *Igf2bp2* sequences contribute to the risk of developing diabetes.

## Materials and Methods

### Animals

All studies on animal tissues were approved by the University of Cambridge Ethical Review Committee and conducted according to the Home Office Animals (Scientific Procedures UK) Act, 1986.

### Cell culture

NIH3T3, 3T3-L1 and HEK293 cells were from ECACC (http://www.hpacultures.org.uk/) and BOSC293 cells from ATCC (http://www.atcc.org/). NIH3T3 and 3T3-L1 cells were grown in Dulbecco's modified Eagle's medium (DMEM) supplemented with 10% newborn calf serum. HEK293 and BOSC293 cells were propagated in DMEM supplemented with 10% fetal bovine serum. All cells were cultured in a humidified incubator with 5% CO_2_ at 37°C.

### Retroviral constructs and site-directed mutagenesis

Oligonucleotide sequences of knockdown constructs and PCR primers are given in Table 1. Complementary oligonucleotides for knockdown shRNA constructs were designed using Dharmacon's siDESIGN web tool with BamHI and EcoRI ends, and annealed in 10 mM Tris-HCl (pH 7.4), 50 mM NaCl. Annealed sequences sh1-F/sh1-R, sh2-F/sh2-R, Scr-F/Scr-R were cloned into the retroviral vector pSIREN-RetroQ (Clontech).

**Table 1:** Sequences of oligonucleotides and primers.

sh1-F	GATCCGGGTAGACATCCACAGAAATTCAAGAGATTTCTGTGGATGTCTACCCTTTTTTCTCGAGG
sh1-R	AATTCCTCGAGAAAAAAGGGTAGACATCCACAGAAATCTCTTGAATTTCTGTGGATGTCTACCCG
sh2-F	GATCCTGACAAGAGAAGAGGCAAATTCAAGAGATTTGCCTCTTCTCTTGTCATTTTTTCTCGAGG
sh2-R	AATTCCTCGAGAAAAAATGACAAGAGAAGAGGCAAATCTCTTGAATTTGCCTCTTCTCTTGTCAG
Scr-F	GATCCGTGCGCTGCTGGTGCCAACTTCAAGAGAGTTGGCACCAGCAGCGCACTTTTTTCTCGAGG
Scr-R	AATTCCTCGAGAAAAAAGTGCGCTGCTGGTGCCAACTCTCTTGAAGTTGGCACCAGCAGCGCACG
IGF2BP-Retro-F	CCCGAGATCTATGATGAACAAGCTGTACA
IGF2BP2-Retro-R	AAATGAATTCTTACTTGCTGCGCTGTGG
BP2-Kozak-F	TTTTAGATCTGCCACCATGATGAACAAGCTG
BP2-1/2Ile-F	TTTTAGATCTGCCACCATCATCAACAAGCTGTACATTGGGAACCTG
sBP2-F	CCCGAGATCTAAAATCATGGAAGTTGAC
BP2-UTR-F	TTTTAGATCTCCACGCGTCCGC
BP2-UTR-R	TTTTGAATTCGCTTTGAGCATGTTCAC
BP2-69Ile-F	GAATTGCATGGGAAAATCATCGAAGTTGACTACTCAGTCTC
BP2-69Ile-R	GAGACTGAGTAGTCAACTTCGATGATTTTCCCATGCAATTC.
BP2-mKozak69-F	ATGGGAAACTTATGCAAGTTGACTACTCAGTCTCTAAAAAGCTAAGGAGC
BP2-mKozak69-R	AACTTGCATAAGTTTCCCATGCAATTCCACTTTACCCGAGAG
FS68-F	GGGTAAAGTGGAATTGCATGGAAAATCATGGAAGTTGACT
FS68-R	AGTCAACTTCCATGATTTTCCATGCAATTCCACTTTACCC
BP2-RACE-Outer	CCTTTCCGATGATGGCACCAA
BP2-RACE-Inner	GGCGAATTCGGGATGTAGGAAATCTTGAAGG.

All overexpression and mutagenesis constructs contained restriction sites BglII and EcoRI, and were ligated into pBabe-puro digested with BamHI and EcoRI. Full length mouse IGF2BP2 coding sequence was amplified from clone 5354659 (Geneservice) using primers IGF2BP-Retro-F and IGF2BP2-Retro-R (*Cds* construct). In this construct the 5′ and 3′UTR sequences are derived from the vector polylinker. To make the construct that included parts of the endogenous *Igf2bp2* 5′UTR and 3′UTR (*UTR construct*) primers BP2-UTR-F and BP2-UTR-R were used. To add an optimised Kozak sequence upstream of the AUG start codon (*Kozak1/2* construct), forward primer BP2-Kozak-F was used instead. To mutate the first two Met codons in the coding sequence (*1/2Ile* construct), forward primer BP2-1/2Ile-F was used. To create a 5′ truncated construct (*Δ5′*), forward primer sBP2-F was used.

Three other constructs were generated by site-directed mutagenesis using Stratagene's QuikChange Lightning Site-Directed Mutagenesis kit according to the manufacturer's protocol. BP2-Kozak/pBabe-puro was used as the template. The internal Met69 was mutated to Ile (*69Ile* construct) using primers BP2-69Ile-F and BP2-69Ile-R. The Kozak sequence surrounding Met69 was mutated using primers BP2-mKozak69-F and BP2-mKozak69-R (*mutKozak69* construct). A single nucleotide deletion at nt198 (Δ*nt198* construct) was created using primers FS68-F and FS68-R.

### Cell transfection and retroviral infection

Retroviral constructs were transfected into BOSC293 packaging cells using Lipofectamine 2000 (Invitrogen). Retrovirus was harvested after 48 h and used with polybrene (16 µg/ml) to infect 3T3-L1 cells. Cells expressing the constructs were subsequently selected by adding puromycin to the final concentration of 4 µg/ml.

### Western blotting

Mouse and rat tissues were removed and snap frozen immediately after sacrifice. Tissues were later thawed on ice, and homogenized in ice-cold lysis buffer (50 mM HEPES pH 7.4, 150 mM NaCl, 10 mM EDTA, 30 mM NaF, 10 mM Na_4_P_2_O_7_, 1% Triton X-100, 1 mM Na_3_VO_4_, 0.5% v/v protease inhibitor cocktail; 100 mg tissue/ml lysis buffer) using an Eppendorf-fitted pestle-homogenizer. Cultured cells were washed with PBS and solubilised in lysis buffer. Lysates were cleared by centrifugation for 5 min at 16000 g to remove cell debris. Extracted proteins were mixed with 4× loading buffer (200 mM Tris-HCl pH 6.8, 4% SDS, 40% glycerol, 100 mM DTT, 0.08% w/v bromophenol blue) and heated at 95°C for 5 min before loading onto SDS-polyacrylamide gels. Proteins were transferred to PVDF membranes which were blocked in TBS containing 5% (w/v) dried milk before incubation with antibody. An antibody against human IGF2BP2 was obtained from Abnova. Rabbit polyclonal anti-peptide antibodies were generated against the C-terminal QHQKGQSNLAQARRK, QEQRYPQGVAPQRSK, and QQKALQSGPPQSRRK sequences of mouse IGF2BP1, IGF2BP2 and IGF2BP3 respectively (CRB Ltd). Primary antibodies were used at a dilution of 1/10000 and peroxidase-labelled polyclonal goat secondary antibodies (Dako) at a dilution of 1/1000. The blots were developed using enhanced chemiluminescence reagents in accordance with the manufacturer's instructions (Pierce).

### 5′RLM-RACE

5′ RNA ligase mediated rapid amplification of cDNA ends (5′ RLM-RACE) was carried out using Ambion's FirstChoice RLM-RACE kit according to the manufacturer's protocol. Total RNA from human HEK293 fibroblasts, mouse 3T3-L1 preadipocytes and rat placenta was used as templates. For the first and second rounds of RACE-PCR, IGF2BP2-specific primers BP2-RACE-Outer and BP2-RACE-Inner were used.
